# The limits of sportswashing. How the 2022 FIFA World Cup affected attitudes about Qatar

**DOI:** 10.1371/journal.pone.0308702

**Published:** 2024-08-16

**Authors:** Johannes Gerschewski, Heiko Giebler, Sebastian Hellmeier, Eda Keremoğlu, Michael Zürn

**Affiliations:** 1 Research Department “Global Governance”, WZB Berlin Social Science Center, Berlin, Germany; 2 Cluster of Excellence “Contestations of the Liberal Script”, Freie Universität Berlin, Berlin, Germany; 3 Research Department “Transformations of Democracy”, WZB Berlin Social Science Center, Berlin, Germany; 4 Department of Politics and Public Administration, University of Konstanz, Konstanz, Germany; Universität Hamburg: Universitat Hamburg, GERMANY

## Abstract

Non-democratic regimes have increasingly been hosting major sports events to boost their visibility and image abroad, which sparked debates about the potential for “sportswashing”. Using the case of the 2022 FIFA World Cup in Qatar we examine how the framing of the tournament influenced opinions about Qatar abroad. Our pre-registered survey experiment with more than 14,000 respondents in eight European countries conducted before the tournament shows that framing it in light of human rights issues in Qatar leads to more negative attitudes towards the host of the World Cup. In contrast, frames emphasizing Qatar’s organizational capacity improve respondents’ attitudes. The heterogeneity of effects across countries highlights the relevance of the national information environment for the effects of major sports events on public opinion. These findings suggest that critical media coverage could potentially mitigate sportswashing efforts while uncritical coverage can increase the legitimacy of autocracies.

## Introduction

Major cultural and sports events provide host nations with a unique opportunity to present themselves in front of the world and shape how a global audience perceives them. While political leaders might be motivated by domestic reasons as well [1–3], authoritarian regimes have seized such opportunities in the past aiming to improve their international reputation. Most infamously, the Olympic Games in Berlin in 1936 were a massive propaganda event in which the Nazis not only displayed their capacity to organize such big events but also propagated their vision of racial supremacy. Argentina hosted the 1978 FIFA World Cup, with the military junta announcing a peaceful tournament while the regime’s major torture center was only several blocks away from the stadium. Recently, non-democratic countries have increasingly hosted major sports events [4]. China, for example, held the Olympic Summer Games in 2008, and the Winter Games took place in Putin’s hometown Sochi. Formula 1 races take place in Bahrain, Saudi Arabia, the United Arab Emirates, and Singapore. Saudi Arabia has been especially active over the last years, buying major influence into golf and professional wrestling and now trying to establish an internationally competitive football league [5]. Looking only at hosts of the Olympic Summer or Winter Games as well as hosts of the FIFA Men’s World Cup and applying regime classifications by the Varieties of Democracy Project [6], three hosts from the period of 1945 to 2000 are classified as closed autocracies. Since 2000, and taking into account already selected hosts, we already count five closed autocracies. Interestingly, this trend ties in with the proclaimed “end of liberal hegemony” [7] and a “third wave of autocratization” [8].

In line with this trend, the 2022 FIFA World Cup was hosted by Qatar, a country in the global bottom 20% in terms of democratic quality but among the top ten richest countries in terms of GDP per capita [9]. FIFA’s decision to award the tournament to Qatar sparked strong but very different reactions. Supporters praised the first World Cup in the Arab world and hoped for positive impulses for the region. Critics pointed to issues like the exploitation of migrant workers, the oppression of women and minorities, and the lack of democracy in the country. While Qatar wanted to use the tournament to generate visibility on the international stage and attract foreign investment, activists aimed to use the World Cup as a focal point to shed light on the country’s poor human rights situation. These controversies speak to the question of whether the use of major sports events helps to “whitewash” a country’s image—a strategy often referred to as “sportswashing”. It can be described as state actors’ activities, often in cooperation with sporting organizations, with the goal of not just hiding information and deceiving, but fostering positive associations with the respective state in the minds of people. It is as a pejorative term, criticizing these activities, often used for the efforts of non-democratic states [10].

Previous research on the economic, political and societal implications of hosting sports events has primarily focused on the domestic level without differentiating between authoritarian and democratic hosts. Organizing major sports events has a significant economic impact. For example, and regardless of the host nation’s regime type, trade for host countries of the Olympic Games increases by 20% on average [11] and tourist arrivals to host countries increase significantly in most cases [12]. At the same time, such mega events generate substantial costs for administration and planning which often leads to a net negative result for host cities [13]. According to some, mega-events should be seen as “loss-making ventures that lack financial sustainability” [14, 1200]. Football tournaments can also contribute to state or nation-building, as people are more trustful of others and more supportive of the state following sports victories in international tournaments [15]. More generally, football affects discrimination, protest dynamics, and even the housing market [16–18].

We know relatively little about the effect of “authoritarian games” and associated sportswashing on the perception of host countries abroad. While it is open to debate whether reputational concerns are the main motivation for autocrats to host international events, recent work on the FIFA World Cup 1978 in Argentina shows that, to prevent negative publicity during the event, the military junta strategically increased repression *before* the games but not *during* the event [4]. Looking to Brazil, in the wake of both the FIFA World Cup 2014 and the Olympic Games in Rio de Janeiro two years later, observers were complaining that the police and military were acting far too harshly and, in some cases illegally, including killings, to ensure that international visitors feel safe during sporting events [19]. In the case of Qatar, pundits coined the term sportswashing, referring to information manipulation by host nations, such as replacing or countering negative content, to improve their image abroad [10, 20]. Qatar even reacted to critique concerning the working conditions under the Kafala system by changing various laws, thereby using the attention created by the World Cup to showcase the willingness to adapt [21]. However, these changes proved to be mainly cosmetic and ineffective [22]—more sportswashing than honest reform.

Sport events draw international attention and affect the reputation of the host abroad, irrespective of governments’ intentions. Being under the spotlight of international audiences can prove to be beneficial but it could also backfire in drawing even more attention to illiberal practices and human rights abuses.

On the one hand, authority and power-holders in general invest a lot to increase their perceived legitimacy with domestic constituencies as well as international observers [23]. Both authoritarian and democratic governments go to great lengths to legitimize their rule domestically [24], but they also care about foreign audiences [25]. Conveying a positive image internationally may benefit authoritarian governments in various ways. Most modern authoritarian governments do not rely any longer on mass repression, but found various ways to “spin” their image in much more subtle ways [26]. A better international standing may boost domestic legitimacy and help alleviate international political pressure [27]. It may also clear the way for further economic cooperation. To improve their reputation abroad, a high-prestige international event such as the World Cup offers a unique opportunity to convey a particular impression [28, 29]. The successful bid generates attention from other countries’ media and foreign audiences, and the way hosts present themselves before and during an event possibly influences how foreign audiences perceive them. Conveying an image of an open, efficient, and high-performing host may help to push illiberal practices and human rights abuses into the background. In this way, hosting major sports events can be seen as an important part of “authoritarian image management” abroad [27].

On the other hand, being under the spotlight of the global community may backfire by drawing even more attention to political wrongdoings. Hosting an international event may expose illiberal practices and human rights abuses and thus invite naming and shaming campaigns by international actors [30]. Controversies around the event may gain in salience and push international audiences to form attitudes towards issues to which they were not exposed before. This politicization process [31] may result in more people developing a critical opinion of the hosting nation by putting illiberal practices under wider public scrutiny. This effect depends upon an open public space for critical reporting and should, therefore especially apply to audiences in more liberal countries with free media as well as higher respect for human rights and critical public discourse. In these environments, whitewashing efforts may prove ineffective or even counter-productive.

In this paper on the FIFA World Cup 2022 in Qatar, we propose a third perspective that combines the two conceivable effects on international audiences and makes them contingent on the frames and messages that dominate public discourse abroad. It assumes that the evaluation of information about sports events in authoritarian regimes varies. Building on framing analysis [32], such variation stems both from what information is presented as well as how information is presented. Crucial factors here are the media structure, the quality of public debates, and the content of reporting about the event. The main question is then which frames dominate public spheres and are decisive for reputational effects for the host in countries abroad.

There is little research on how the FIFA World Cup in Qatar was portrayed in different countries and the same holds for information about more general issues like human rights violations. The recent study by [33] is a notable exception. They show that the World Cup did not improve Qatar’s image but that it had a positive spill-over effect on how Germans view other Arab countries. Another study [34] found that reporting about Qatar in newspapers in three West European countries and the US was much more neutral or even negative than in Chinese newspapers in the year before the tournament in Qatar took place. Similar patterns can be found for reporting on mass protests demanding the overthrow of Hosni Mubarak’s regime in 2011, where Chinese and Arab media were more prone to report favorably about the government, while The Guardian and the International Herald Tribune focused on the opposition movement [35]. Finally, NGOs concerned with human rights (violations) see media attention and information politics as one of their most important tools [36]. As access to media outlets for such NGOs is much easier if there is a high-quality, pluralist media environment, there is more critical information available in such environments.

In sum, while the legitimation and politicization perspectives point to either positive or negative effects, we aim to provide an idea under which circumstances we should expect either positive or negative effects to prevail by pointing to the importance of frames and the media structure in the information receiving countries. This perspective puts a lot of weight on the role of the information environment [37], but leaves room for other (individual-level) factors moderating the effect of major sports events hosted by autocracies on foreign public opinion.

Given conflicting expectations about the effects of hosting major sports events for authoritarian regimes, this study examines data about Qatar’s reputation and the effect of three different frames of Qatar as the host of the FIFA 2022 World Cup using an experimental approach. We first propose that how the event is framed affects individuals’ attitudes about the host. Moreover, we suggest that the dominance of a particular frame in a receiving country is dependent on its media structure. To test this claim empirically, we draw on a large-scale survey experiment fielded just before the beginning of the tournament with more than 14,000 respondents in eight European countries. We prime respondents with vignettes that *(i)* emphasize the human rights situation in Qatar, *(ii)* emphasize the efficient and sustainable organization of the event, or *(iii)* merely mention the occurrence of the tournament. Afterward, we ask for their evaluation of Qatar in its role as the host nation organizing the event. Our findings show that how the tournament is framed affects how people think about the World Cup in Qatar, suggesting that both backlash and whitewashing effects are possible. Thereby, providing a negative frame has a stronger effect in absolute terms than emphasizing efficiency and sustainability. Moreover, we provide tentative evidence that country-level differences in frame resonance and Qatar’s reputation can be attributed to the structure of the media and the public in different countries.

## Results

Our results are based on a large-scale comparative survey experiment that we conducted in cooperation with the survey company Bilendi in eight European countries: Croatia, Hungary, Germany, Italy, Romania, Poland, Sweden, and the United Kingdom. The survey collected data on nearly 2,000 respondents from each country applying quota sampling. We chose these countries to include liberal democracies with a high level of media pluralism and less democratic countries with less free and more oligopolistic media systems. We also varied the expected strength of the men’s football team. In addition, half of the countries did not participate in the World Cup, which should lead to less public interest in the tournament. While the usage of online-access panels—even if they are of high quality such as the ones we used, and as we only look at eight countries—calls for caution concerning generalizability, this country heterogeneity substantially increases the weight of our findings. More information on the case selection can be found below in the Section on Materials and Methods.

The experiment was fielded in the weeks before the World Cup’s kick-off from October 28 to November 18, 2022. We understand this period as a time in which respondents were already exposed to coverage of the tournament, but the event was far from omnipresent. In terms of conservative testing, this constitutes a middle-ground situation. Positive as well as negative information about the World Cup in Qatar and the country itself is not completely new or unknown, but citizens in the different countries were not exposed to news coverage about the event on a daily basis—as was the case during the tournament. We applied quota sampling based on age, gender, education, and place of residence in all eight countries. Whenever applicable, we also use post-stratification weights based on these characteristics, further increasing representativeness within the limits of online access panels. Due to missing values, the sample size drops to roughly 14,000 respondents for the main analyses. The analysis was pre-registered [38] and there are no substantial deviations from the respective pre-analysis plan (see Materials and methods for details).

### Framing the 2022 World Cup in Qatar

To investigate the potential effect of Qatar hosting the 2022 FIFA World Cup on public opinion in foreign countries, we embedded a *framing* experiment in the survey. Respondents were exposed to different “emphasis frames” through short vignettes about the World Cup in which we manipulated how information is presented and its actual content [39]. We devised three different frames about Qatar as the World Cup host: *(i)* a neutral *sports* frame that describes some basic and uncontroversial facts about the tournament, which we assign to a control group, *(ii)* a negative *human rights* frame that emphasizes Qatar’s questionable human rights record, *(iii)* a positive framing that highlights the country’s *efficiency* in the organization of the World Cup. All three frames were based on factual information or pre-existing text bits to increase external validity. While the second frame presents a more negative as well as realistic depiction of Qatar, the third frame emphasizes positive aspects and could qualify as sportswashing. For better readability, we label the frames *(i)* neutral, *(ii)* negative, and *(iii)* positive. The neutral frame serves as the control group in our analyses. The full texts, their sources, and expected effects are summarized in Table 2 in the Methods section below. The questionnaire also included a behavioral outcome measure placed after treatment and additional analysis underlines the validity of our treatment frames (see Table S1 in the S1 File).

Our outcome measure is a latent variable underlying respondents’ agreement with the following three statements which were all measured on a scale from 1 (fully disagree) to 7 (fully agree): *(i)* “The decision to award the FIFA World Cup tournament to Qatar was reasonable”, which asks about the actual decision to hold the event in Qatar. *(ii)* “The FIFA World Cup will be well-organized by Qatar”, which refers to Qatar’s organizational efficiency. *(iii)* “Qatar’s reputation will improve due to hosting the FIFA World Cup”, which asks about the expected effects for Qatar as a hosting nation. We extract the latent variable based on country-specific confirmatory factor analyses (see Table S2 in the S1 File). Our approach ensures a broad perspective on attitudes about Qatar while increasing measurement reliability. Finally, we rescale the latent measure to the original scale of the observable variables. Hence, higher values represent more positive attitudes about Qatar.

**Fig 1:**
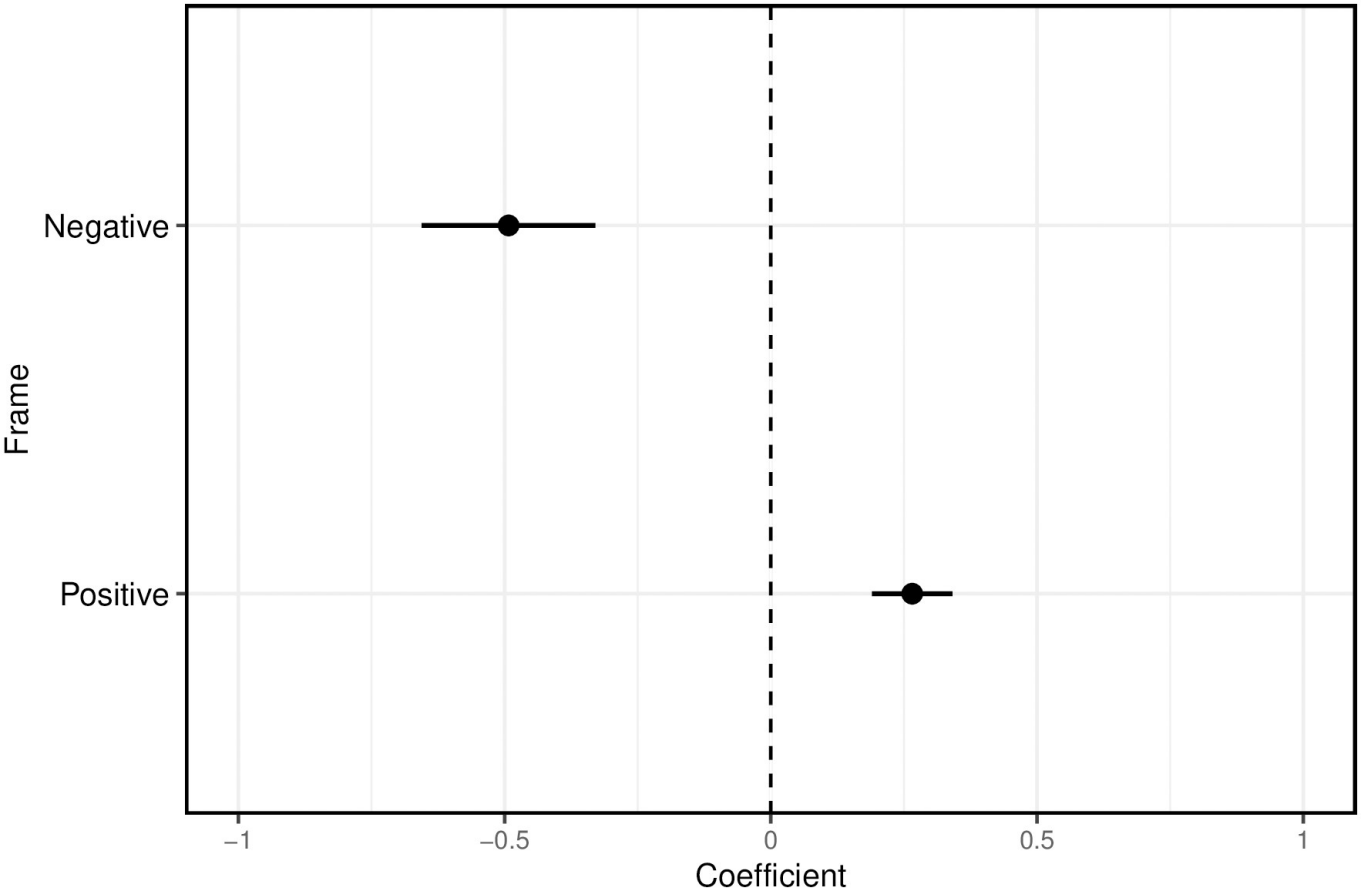
Treatment effects of the positive and negative frames compared to the control group. Estimates based on OLS regression with country dummies and post-stratification weights. Standard errors clustered at the country level (N = 14,017). Outcome: latent measure of attitudes about Qatar underlying respondents’ agreement (1 = fully disagree, 7 = fully agree) with the following three items: *(i)* “The decision to award the FIFA World Cup tournament to Qatar was reasonable”, which asks about the actual decision to hold the event in Qatar. *(ii)* “The FIFA World Cup will be well-organized by Qatar”, which refers to Qatar’s organizational efficiency. *(iii)* “Qatar’s reputation will improve due to hosting the FIFA World Cup.” Figure based on regression results from Model 1 in Table S3 in the S1 File.

The negative and positive frames significantly shape respondents’ attitudes toward Qatar compared to the neutral frame (see also Table S3 in the S1 File). Fig 1 shows substantial treatment effects based on an OLS regression model with the framing treatments as independent variables. The negative human rights frame leads respondents to a significantly worse evaluation of Qatar than the neutral sports frame. By contrast, emphasizing effective organization using a positive frame leads to a more positive assessment. The effects are substantive: The difference in assessing Qatar as a host between the human rights and the efficiency frame is close to three-quarters of a scale point. The dependent variable only has a standard deviation of 1.18, which further underlines the relevance of the identified treatment effects. Finally, the fact that the negative effect is larger is fully in line with the well-known “negativity bias” and loss aversion proposed by socio-psychological and cognitive theories [40].

### Differences across countries

What are the difference between countries? The experiment was carried out simultaneously in eight countries: Croatia, Hungary, Germany, Italy, Romania, Poland, Sweden, and the United Kingdom. All countries are part of Europe, are or have been members of the European Union, and are no traditional allies of Qatar. Yet, these European countries differ significantly on key dimensions that affect the expected “resonance” [41] or effectiveness of our different frames. First, half of the countries did not participate in the tournament. Therefore, one could expect the salience of the World Cup in general and event-related media coverage to be lower in Hungary, Romania, Sweden, and Italy. Second, the information environment varies in important ways. Some countries (Germany and Sweden) have high levels of media freedom and a pluralist media system with a dominance of so-called high quality media. In others, such as Romania and Hungary, there is more state intervention and less pluralism in the media. These countries represent oligopolistic media markets that could inhibit critical reporting about events like the tournament in Qatar [42]. Thirdly, there is ample heterogeneity when it comes to the quality of democracy with Sweden on one end and Hungary, classified as an electoral autocracy, on the other [9]. Finally, there is also substantial variation when it comes to the age of democracy as well as geographic location and associated characteristics.

**Fig 2:**
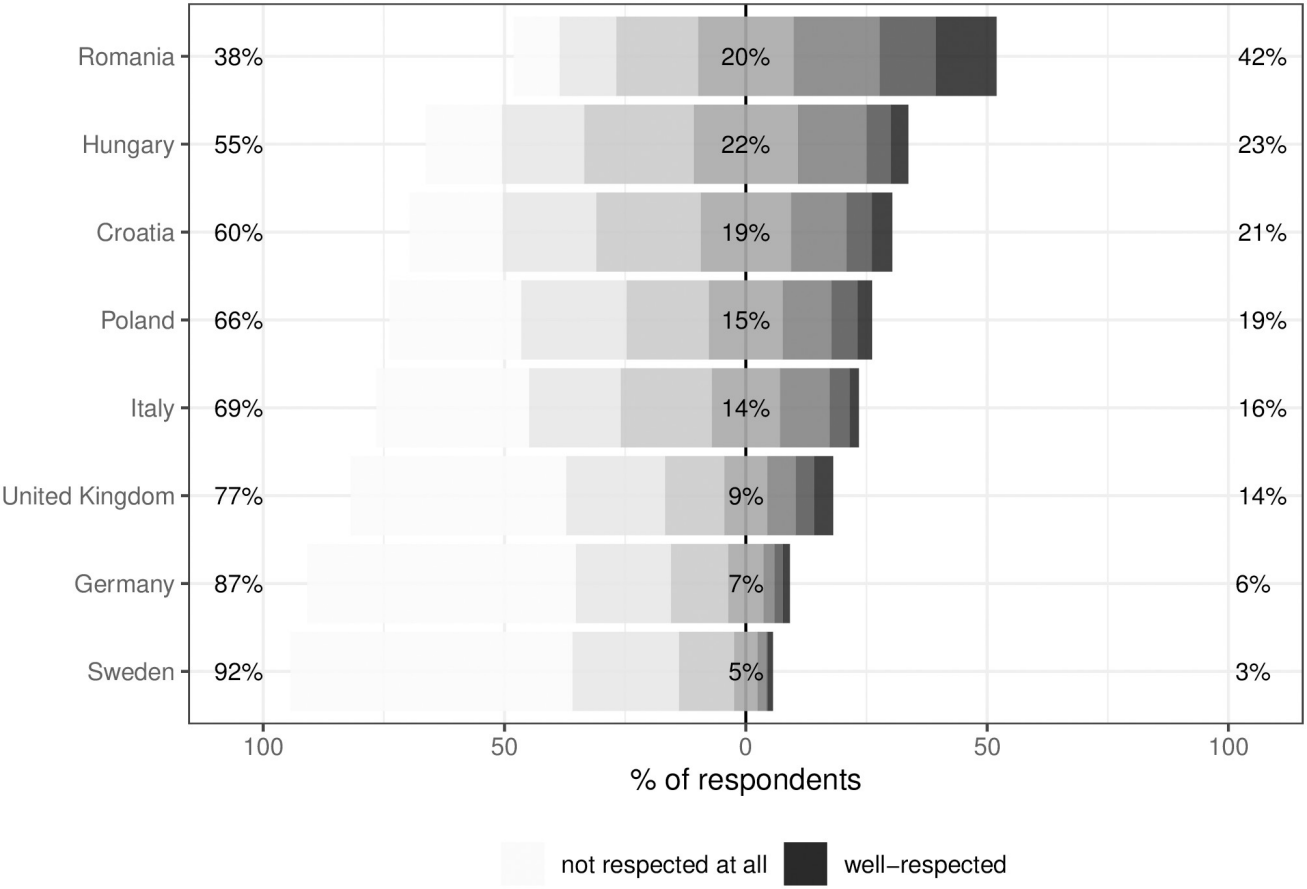
Distribution of responses on the assessment of the human rights situation in Qatar by country. The x-axis plots the share of respondents with negative (left of 0) and positive assessments (right of 0) respectively on a 7-point scale where 1 = “not respected at all” and 7 = “well-respected” for each country (y-axis). Percentages per country indicate the aggregate share of all respondents with a negative (below 4) and with a positive assessment (above 4) respectively. Number of valid responses = 11774. Missing values (“don’t know”, “don’t want to respond”) omitted from the graph.

Descriptive statistics from our survey showcase the variation in attitudes towards Qatar across the eight countries. Among other things, for example, we asked respondents to assess the human rights situation in Qatar before our experimental intervention. According to Fig 2, in Germany and Sweden, around 90% of respondents have a rather negative image of the human rights situation, compared to only about 70% in Italy and Poland. Respondents in the United Kingdom fall somewhere in between. Romania stands out as the only country where the overall assessment of human rights in Qatar is balanced, and a notable share of respondents even evaluate the protection of human rights rather positively. Hungary and Croatia lie in between Poland and Romania. These descriptive findings show that there are enormous differences regarding the human rights reputation of Qatar, which makes it highly likely that there is also considerable cross-country variation in public discourse about the tournament.

**Fig 3:**
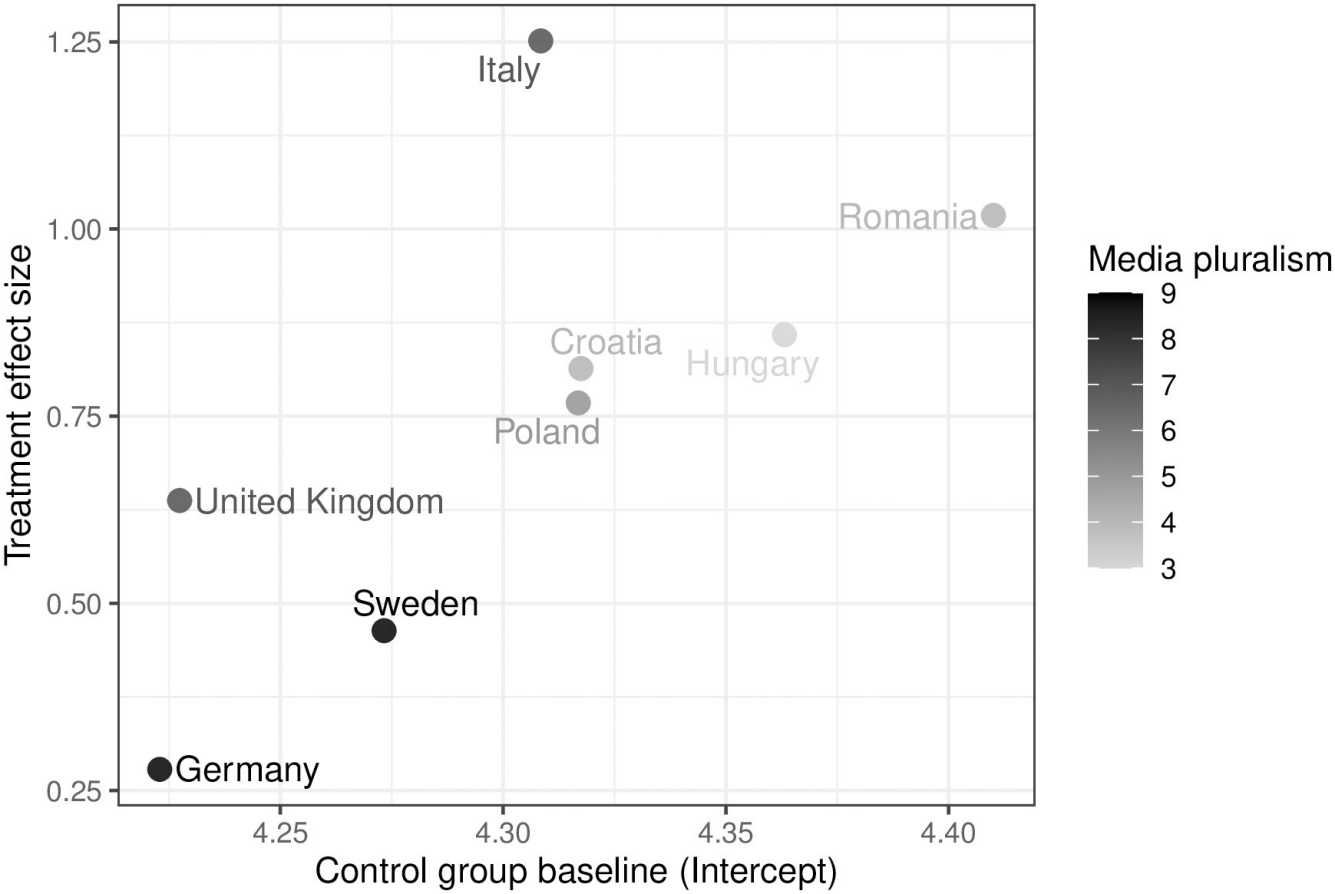
Heterogeneous treatment effects. Heterogeneity of framing effect strength across countries with treatment effect size on the y-axis and the baseline (intercept) of the control group on the x-axis. Data on media pluralism taken from the SGI [42], color-coded according to level of pluralism with light gray indicating lower and dark gray higher levels of media pluralism (1 = lowest, 10 = highest). Effect sizes and figure based on regression results with post-stratification weights from Table S4 in the S1 File.

To assess these differences across the countries in our sample more systematically, we ran the regression analysis separately for each country (see Table S4 in the S1 File). Fig 3 summarizes the findings. On the x-axis, we plot the average attitudes about the World Cup in Qatar for the control group to provide a baseline for each country. The y-axis shows the effect size of our treatments measured by calculating the difference between the effects for the positive and negative frames. This measure tells us how strongly respondents react to the positive and negative frames in each country. The figure shows that there is a correlation between baseline values and effect size—with Italy and the United Kingdom a bit off the imagined line. In Germany and Sweden, where the control groups showed more negative attitudes about Qatar at the time of the interview, the frames have a much smaller effect compared to Romania and Hungary, where respondents had a much more positive image of Qatar and also its human rights track record (see above). The identified country differences are quite substantial.

As stated above, we argued that the media and information environment are key in shaping public discourse about authoritarian host countries of major sports events. Therefore, we also include information on levels of media pluralism in each country in Fig 3. The data comes from the Sustainable Governance Indicators (SGI) 2022 [42] and ranks media systems from oligopolistic to diversified or public ownership on a scale from 1 to 10. The resulting figure shows a tendency that framing effects are stronger in countries with oligopolistic media ownership structures. At the same time, the countries that did not qualify for the World Cup (Sweden, Italy, Hungary, Romania) also show a tendency for greater effect sizes compared to participating countries with similar levels of media pluralism. Again, Italy is an outlier as we would expect a smaller effect similar to the other non-participating countries. Thinking about how important football is in Italy, this could be explained by the country’s unexpected failure to qualify for the World Cup resulting in low public interest in the tournament. Taken together, this is initial evidence for our claim about the relevance of the information environment in shaping public discourse about “authoritarian games”. Where interest (participating countries) and media pluralism are high, respondents are more critical of Qatar and are less likely to be swayed by positive or negative frames. For the negative frame, this is probably due to pre-existing knowledge about the situation in Qatar. At the same time, sportswashing—the positive frame—is less successful as recipients are highly likely to have received deviating and more accurate information as well. While the eight countries clearly differ on several other dimensions, e.g., wealth, government composition, or socialist past, it seems striking that the media and information environment argument, with the exception of Italy and to a lesser degree the United Kingdom, explains the identified pattern well. There is some additional tentative evidence emphasizing the importance of a high-quality, pluralist media environment. Fig 2, suggests that countries with a more plural media environment assess the human rights situation in Qatar more critically. In a way, we already see exposure toinformation at work here—something we recreate experimentally with our frames. Further analyses also show that respondents living in environments with less plural media are more likely to be unable to assess the human rights situation in Qatar at all and that treatment effects are much larger for respondents unable to assess the human rights situation (see Table S5 in the S1 File). While only indirect in nature, we interpret this as additional evidence that oligopolistic media environments provide less information and space for pluralistic discourse on Qatar, and that this makes citizens more responsive to our information treatments. Consequently, these citizens would also be more responsive to sportswashing or informational countermeasures.

We are aware that our cross-country comparison with eight cases does not allow for strong causal claims regarding the role of the information environment. There might be other factors not discussed in this paper that also moderate the treatment effects or drive more general attitudes about Qatar in different countries. Moreover, the quality of the information environment is associated with other aspects like the quality of democracy or the protection of civil rights. At the same time, looking at the strength of the treatment effects, it seems highly plausible that information plays a decisive role.

### Robustness of main findings and additional subgroup analysis

We probe the robustness of our main findings by running additional tests, including several subgroup analyses. In a first step, we re-run our main model controlling for compositional effects by adding variables such as political interest, education levels or interest in football (see Table S2 in the S1 File). Second, we re-run our main model using a multilevel model where respondents are nested in countries (see Table S6 in the S1 File). Third, we run models using the three manifest response variables as outcome variables (see Table S7 in the S1 File). Fourth, as specified in the pre-analysis plan, we re-run our main model using dummy coding for our outcome measure estimating non-linear regression models (see Table S8 in the S1 File). Fifth, we run a regression model specifying an interaction between country dummies and the treatment variables as an alternative test of country-level heterogeneity (see Table S9 in the S1 File). None of these robustness checks leads to different conclusions.

**Fig 4:**
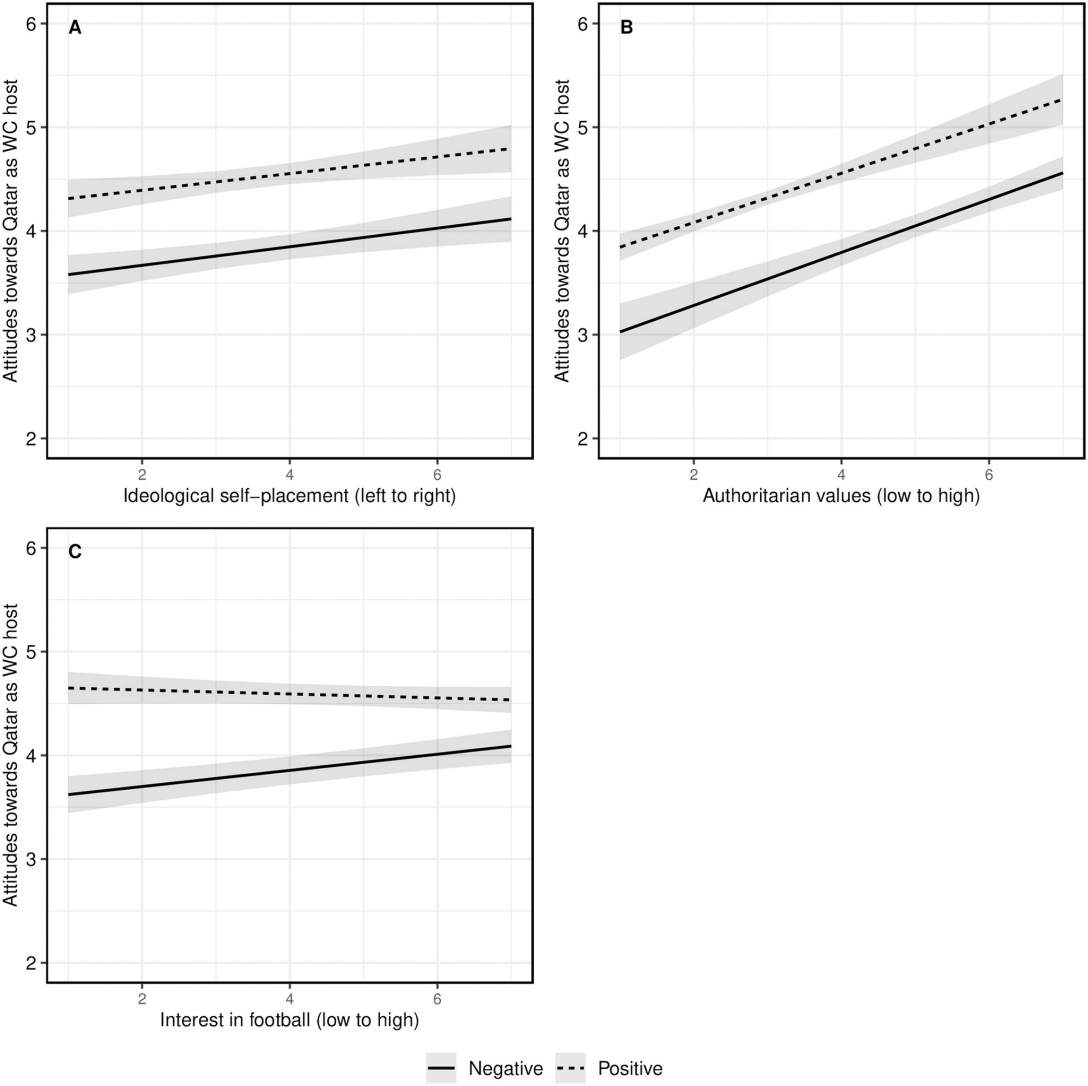
Assessment of heterogeneous treatment effects at the individual level. Predicted values of attitudes towards Qatar as the World Cup host (y-axis) by respondents’ individual characteristics (on respective x-axes): **A:** Ideological self-placement from left to right. **B:** Authoritarian values from low to high. **C:** General interest in football from low to high. Predicted values based on OLS regressions with post-stratification weight, country dummies, and clustered standard errors on the country level. Figure based on regression results from Table S10 in the S1 File.

Next, we examined effect heterogeneity at the individual level, as the perception of the different frames could differ depending on respondents’ ideological stance or knowledge about Qatar and the World Cup. Fig 4 displays the effects of the negative human rights and the positive efficiency frames across subgroups based on estimated interactions. The first two variables, political ideology (Panel A) and authoritarian attitudes (Panel B), were part of this study’s pre-registration. We expected that right-wing and authoritarian individuals would give less weight to information about the lack of democratic governance and human rights protection in Qatar. While it is the case that individuals on the right of the ideological spectrum and with more authoritarian values tended to assess Qatar more positively, we did not find significant differences in treatment effects (see Table S10 and section S10 in the S1 File for more information on the measures).

We also tested effect heterogeneity for one additional variables, considering that the information we present in our experiment may not be new to all respondents, which should decrease treatment effects [43]. Panel C in Fig 4 shows predicted values for the interaction between the two treatment frames and general interest in football. We find significant subgroup differences depending on respondents’ interest in football. More precisely, the negative human rights frame has smaller effects on respondents highly interested in football. This finding could be because respondents are better informed about the World Cup, and reading about the human rights situation in Qatar is no new information. However, the fact that the efficiency frame effect does not diminish with increasing interest in football could suggest that these respondents with a high interest in football engage in motivated reasoning and discount negative reporting about an event they have been looking forward to [44]. Nevertheless, we still see a significant difference between the two treatment groups for all levels of interest in football. Overall, these additional analyses show that the treatment effects are stable and do not vary much due to individual-level characteristics (see Table S10 in the S1 File). This further increases our confidence that country-level variation is not driven by these individual-level characteristics.

## Discussion

While domestic motivations to host the tournament might have played a role as well, the FIFA World Cup 2022 presented the Qatari government with a unique opportunity to present itself on the international stage. Our study investigated whether such a major sports event contributed to authoritarian image whitewashing abroad. In a survey experiment, we assessed whether positive and negative frames influenced how respondents in eight European countries perceived the hosting of the World Cup in Qatar. Our study presents three key findings which are fully in line with our pre-registration of the study. First, it demonstrates the importance of framing effects around the FIFA World Cup in Qatar, emphasizing that exposure to particular information can shift individuals’ approval of authoritarian hosts. Second, whether authoritarian games have politicization or legitimation effects depends on the availability of critical information about human rights abuses, which can mediate the framing effect. Third, frame effects vary widely between countries. Respondents in countries with a pluralist and free information environment seem to be much less receptive to negative frames compared to respondents living in countries with oligopolistic media. They have been exposed to negative information about the human rights record from the beginning and therefore are relatively immune to further frames in this direction.

While the small number of countries does no allow a robust test for macro-level effects, our third finding suggests that a country’s political and media landscape moderates how and which information is received. For respondents living in more liberal states with a plural media landscape, politicization is the predominant effect. People in these countries have a negative and relatively stable view of the games in Qatar. Here, additional information has little effect independent of the frame. Respondents living in less liberal states, on the other hand, perceive Qatar as much more positive. For these individuals, the differential effect between negative and positive frames also remains significant (Fig 3). In general, audiences abroad will likely change their attitudes towards authoritarian hosts of major sports events in response to the additional information about the host and the games. Whether the effects are positive or negative, however, depends mainly on features of the society they live in.

Authoritarian hosts cannot assume that sportswashing will work everywhere. The effect of authoritarian games on the host’s reputation depends on frames and media structures on the information-receiving side that are not fully under the control of the organizer. In addition to boycotting and transforming ways of participation [45], a third strategy to oppose sportswashing simply relates to making factual information about host countries available. Critical publics and media do not simply accept decisions by the FIFA and the International Olympic Committee. Hence, the effect of authoritarian games abroad depends on the quality and sentiment of media reporting. At the same time, reputation management by hosting authoritarian games works better in states with oligopolistic media structures often associated with authoritarian states. Against this background, the reported rise in reputation and status that Qatar experienced in the Arab world does not come as a surprise.

Nevertheless, our study has a few limitations that underscore the need for further research in this area. First, while our individual-level analyses with randomized treatment assignments allow for the identification of causal effects of framing on attitudes, we can only provide correlational evidence for our analyses on country-level contextual factors with eight observations. Although we cannot rule out all alternative explanations, such as differences in liberal attitudes across societies, we made a clear case for the importance of media pluralism and enough critical coverage of authoritarian hosts. As for the majority of citizens, the media is the only point of contact with the host country, we are convinced that public discourse plays a major role. Still, other contextual factors could play a role and should be taken into account if the number of countries allows it. Second, we cannot know whether the effects are durable and whether the different frames have behavioral consequences besides attitudinal change. Third, our sample is limited to a subset of European countries, and we cannot know if our results travel to other contexts. In particular, how audiences in Qatar’s neighboring countries perceive the host nation remains an open question. It seems likely that this may differ depending on regional (power) politics and dynamics. Fourth, we do not test the effects of competitive frames—for example, provide positive and negative information about the tournament in Qatar to respondents—which is sometimes described as a more realistic representation of citizens’ reality [46]. All in all, further research on the perception of authoritarian host countries, especially in non-democracies, is needed to understand whether and to what extent the organization of major sports events benefits autocrats abroad.

To sum up, based on an experimental study, our findings imply that, if autocratic hosts manage to get their message out, they are indeed able to create a more positive image internationally. However, if negative or non-sportswashed information is available, Qatar’s reputation suffers substantially. This puts a lot of emphasis on media quality on the one hand and social media as well as fake news on the other—even more so as we can show that the effects of a negative frame seem to be stronger in information environments of lower quality. With another FIFA World Cup now very likely taking place in Saudi Arabia—again a political regime with a very bad human rights record—our study provides strong evidence that fostering a critical debate about the situation in Saudi Arabia becomes crucial, especially in countries with lower quality and less pluralistic information environments.

Our study has important policy implications that can help mitigate sportswashing with the help of events like the 2022 World Cup in Qatar. First, our study underlines the importance of media pluralism and critical public discourse in understanding sportswashing. In countries with oligopolistic media system, independent actors such as international human rights organisations could still counter sportswashing effects if they raise awareness of host countries’ human rights records and engage in independent reporting. The latter not only pertains to reporting on host countries, but also to calling out international sports governing bodies if they do not rely on criteria such as candidate countries’ respect for human rights and democracy when awarding sports events. Second, the frequent demand that sport and sporting events should not be about politics or the like, but only about sport, should not be conceded—neither by participants, the media, (I)NGOs, nor by foreign governments. Our findings clearly show that a lack of contextualizing political information surrounding sports events makes it much more likely that autocracies are able to increase their reputation abroad. Finally, an independent monitoring body to oversee and report on the compliance of host countries with these human rights standards before, during, and after the event could be established. Such a body could then further ensure that actual information on host countries is available. While sportswashing does not take root everywhere as our study shows, acknowledging and addressing the politics surrounding major sports events as one way of polishing autocracies’ image on the international stage is crucial.

## Materials and methods

### Ethical compliance

The research design and the questionnaire were approved by the WZB Berlin Social Science Center Research Ethics Committee (review 2022/9/174) prior to conducting the study. It is important to highlight that the study does not involve deception, given that the different framing treatments are based on actual coverage of the World Cup disseminated by various public sources.

### Pre-registration

They analysis was pre-registered [38]. However, as this endeavor is part of a much larger research project, some additional and exploratory analyses named in Pre-registration are not part of this study. Furthermore, for the sake of a more accessible presentation, the main analysis uses a latent variable and not each response variable separately as originally stated. However, results for the latter approach are presented as robustness checks and there are no relevant differences. All other analyses are fully in line with the pre-registration.

### Country selection, sample, and data

This study is part of a larger project that investigates the potential effects of the FIFA World Cup in Qatar in various ways and from different angles. We developed a selection strategy to maximize generalizability by selecting countries that differ on important dimensions. We have also limited the geographical focus to Europe ensuring some comparability. We are fully aware that any analysis relying on public opinion data from eight countries has to be conscious concerning its limitations. At the same time, we selected our country cases carefully to maximize our studies potential.

Our selection of European countries was made in an attempt to maximize variation on three dimensions: *(i)* a country’s level of liberal democracy, including pluralism in a country’s media environment *(ii)* the strength of each country’s national football team to approximate the relevance of football, and *(iii)* whether the country participated in the World Cup to capture public and media interest in the event.

**Fig 5:**
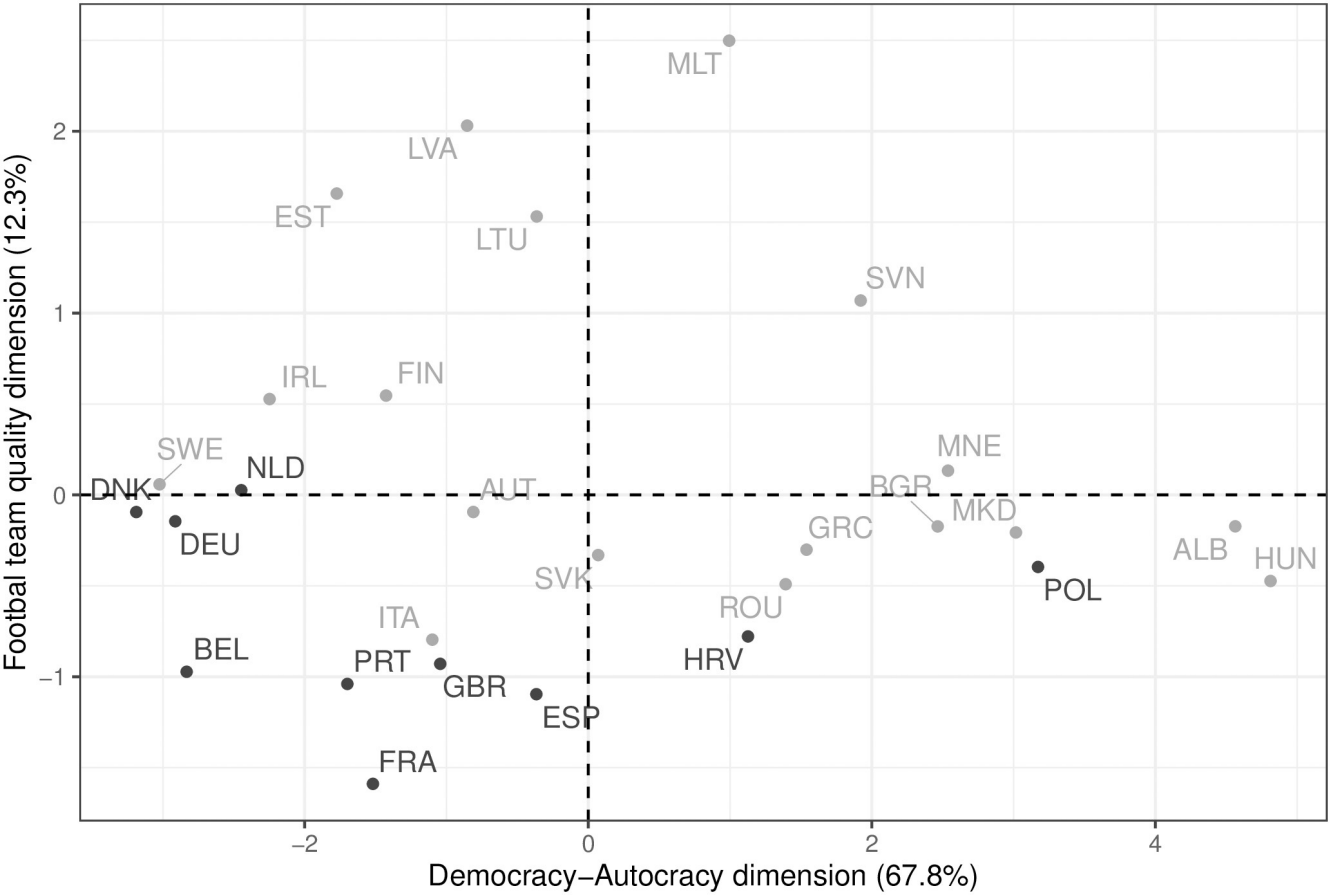
Case selection. Principal component analysis along *(i)* the level of democracy (x-axis) and *(ii)* the quality of the football team (y-axis) with percentages of explained variance in parantheses. Countries participating in the 2022 FIFA World Cup are color-coded in dark gray, non-participants in light gray.

To identify suitable countries, we run a Principal Component Analysis (PCA) with information on the level of democracy, media freedom, freedom of expression, human rights protection and the current FIFA ranking. The data comes from the Varieties of Democracy Project [6, 47, 48]. The PCA reduces dimensionality by estimating latent variables that are linear combinations from all input variables and capture as much variation as possible. Fig 5 summarizes the results by locating all countries with available data along two dimensions that capture the level of democracy as well as pluralism and the quality of each country’s national men’s football team. The latter dimension, taken together with the fact whether a country is participating in the tournament or not, can help to identify the degree to which the country is exposed to the event and Qatar’s positive framing of the tournament.

Participating countries are marked in dark gray, and countries which did not qualify are presented in light gray. Based on these numbers, we select four pairs of countries along the democracy-autocracy continuum with one participant and one non-participant country while keeping the football quality dimension as stable as possible for each pair. In addition, to ensure data quality, we took into account in which countries there are high-quality online-access panels. Table 1 presents the selected pairs. Here, pairs are sorted based on the quality of the country’s media system and democracy as well as the human rights situation. Germany and Sweden form the top pair while Poland and Hungary constitute the pair with the worst situation.

**Table 1:** Overview of selected countries.

Pair	Participating country	Non-participating country
1	Germany	Sweden
2	UK	Italy
3	Croatia	Romania
4	Poland	Hungary

Pairs of countries participating and not participating in the World Cup were selected based on similarity in level of democracy and quality of the football team.

In each country, we administered an online survey using quota sampling in cooperation with the survey company Bilendi. Bilendi not only took care of scripting the survey, but was also responsible for translating the English master questionnaire—with the exception of the German translation which was done by the authors. Bilendi contracted professional translators for each language and each translation was validated by a second translator (four-eyes principle).

Within the limits of high-quality online-access panels, each country sample was a representative population sample of the adult population based on age, gender, education, and place of residence. Overall, we originally surveyed 15,930 individuals in all eight countries. For our main analyses, we removed those participants from our sample who spent less than five minutes on the questionnaire and those who failed attention checks, which leaves us with 14,626 respondents. This rule to remove speeders and inattentive respondents was specified in the pre-analysis plan. The number of observations in our regression analysis is slightly lower due to missingness (“don’t know” and “don’t want to respond”) on all variables used to measure our outcome variable (N = 14,017). The survey was fielded between October 28 and November 18, 2022 and respondents consented by participating in the online survey.

### Measurement

#### Outcome variable and manifest items

Our main outcome variable constitutes a latent variable and it is based on three items measuring respondents’ attitudes about Qatar and the FIFA World Cup. We asked respondents whether or not they agreed with the following statements on a scale from 1 (fully disagree) to 7 (fully agree): (1) The decision to award the FIFA World Cup tournament to Qatar was reasonable; (2) the FIFA World Cup will be well-organized by Qatar; (3) Qatar’s reputation will improve due to hosting the FIFA World Cup. The three measures capture different aspects of Qatar hosting the World Cup. The first item is about the past (award decision), whereas the other two items concern the near (organization of the tournament) and mid-term future (reputation effects). Item order was randomized for each respondent. We run country-specific confirmatory factor analyses to confirm that all three statements tap into the same underlying dimension. We collapse respondents’ answers into a latent measure of attitudes about Qatar as the host of the FIFA World Cup by extracting the latent variable. Cronbach’s Alpha values are very high and they range between 0.78 and 0.86. Running separate models to extract the factors constitutes a more conservative test as it allows for structural differences between countries. More information can be found in section S2 in the S1 File. The latent variable is rescaled to the original seven-point scale.

#### Treatments and randomization

In our survey, we rely on a *framing* experiment. As we are interested in citizens’ attitudes concerning an autocratic regime such as Qatar hosting the tournament and their evaluations thereof, providing different information frames constitutes a very promising approach [49]. We implement emphasis frames which refer to differences not only in how information is presented but also in the actual content [39]. We use three different frames to provide information about the tournament: a positive efficiency frame, a neutral sports frame, and a negative human rights frame. We use actual information or pre-existing text bits for all three frames to increase external validity. This is crucial when running survey experiments in general and framing experiments specifically [50]. We randomly assign our three issue frames to respondents. Respondents who receive the sports frame form our control group as they are presented with neutral information about the World Cup taken from Wikipedia. The efficiency frame constitutes our first treatment, which we label “positive”, as respondents in this group receive information about Qatar’s efficient organization of the tournament and sustainability efforts. Here, the frame is inspired by information from the Qatar Ministry of Tourism. Our second treatment, labeled “negative”, is based on the human rights frame, which presents respondents with information highlighting Qatar’s illiberal practices and lack of respect for human and workers’ rights as presented in an original investigation by The Guardian. Table 2 gives the wording of each frame that respondents from our control and the two treatment groups respectively, read before proceeding with our survey. Randomization was carried out by the survey company and applied country by country. There were no issues with the randomization process.

**Table 2:** Experimental setup and issue frames.

Treatment group	Issue frame
Sports (neutral)	The twenty-second FIFA World Cup takes place from November 20th to December 18th. The tournament organizer, which takes place every four years, is the Emirate of Qatar. Due to the weather conditions in Qatar, the tournament will not take place as usual in the summer months. Thirty-two nations will compete in the tournament and there will be all in all sixty-four games. [Source: Wikipedia]
Human rights (negative)	The staging of the FIFA World Cup in Qatar has caused some controversy in the run-up to the event. For example, the working conditions for foreign workers were much criticized. According to reports, about 6,500 migrant workers have died since 2010 while building infrastructure for the World Cup. In addition, homosexuality is still prohibited by law in Qatar, women are strongly discriminated against and only a minority of citizens has the right to vote [Source: Reporting by The Guardian]
Efficiency (positive)	Qatar is the first Arab country to organize a FIFA World Cup which is supposed to spur economic growth in the region. Among other things, Qatar built Stadion 974, one of the most modern football stadiums in the world. It consists largely of recycled material and can be dismantled after the end of the World Cup. The new stadiums are so well connected that fans can watch multiple games in one day and 20,000 volunteers will help across areas from health and safety to medical and language services. [Source: Qatar Ministry of Tourism]

Respondents were randomly assigned to one of the three treatment groups and were asked to read the respective frame.

### Statistical analysis

First, we estimate the causal effects of the different frames on respondents’ assessment of Qatar as the host of the World Cup measured as a latent variable. Therefore, we ran OLS regressions with the randomly assigned frames as the independent variables and our latent variable as the dependent variable. Our main independent variable is a factor variable representing the treatment groups to measure the frames’ effects. As specified in the pre-analysis plan, we use the standard p<.05 criteria for determining if the results are significantly different from those expected if the null hypothesis were correct. The main regression model only includes our treatment variable and country dummies to absorb all country-level factors. Standard errors are clustered at the country level as well. All regressions include post-stratification weights on the individual level to further increase representativeness. Furthermore, we added weights on the country level in a way that assigns identical weights to the sum of all respondents of the country. We estimated the weights based on an iterative proportional fitting algorithm [51]. The main regression table in the S1 File shows a significant effect for the two framing treatments compared to the control group (sports/neutral framing). Given that we randomize treatment assignment, we do not need to include variables to adjust for confounding. Nevertheless, we re-ran our main regression model, including a series of individual-level controls such as gender, age, education, political interest, left-right self-placement, and interest in football, obtaining near-identical results. We also conducted several additional robustness checks (see above) and all the information can be found in the S1 File.

## Supporting information

S1 File Supporting information. (PDF)
