# Supplementary material for: The limits of sportswashing. How the 2022 FIFA World Cup affected attitudes about Qatar
Source: PLoS One. 2024 Aug 16;19(8):e0308702. doi: 10.1371/journal.pone.0308702 (PMC11329124; doi:10.1371/journal.pone.0308702)
Supplement: S1 File — (PDF) [file pone.0308702.s001.pdf]

Supporting Information for

The Limits of Sportswashing. How the 2022 FIFA World Cup Affected Attitudes about Qatar

Johannes Gerschewski[1], Heiko Giebler[1,2], Sebastian Hellmeier[1], Eda Keremoğlu[3], and Michael Zürn[1,2]

[1]WZB Berlin Social Science Center
[2]Freie Universität Berlin
[3]University of Konstanz

This PDF file includes:

Supporting Text
Tables S1 to S10

S1 Behavioral outcome measure

The questionnaire also included a behavioral outcome measure. We offered respondents to learn more about the human rights situation in Qatar after the experiment. If they agreed, we showed them a short summary of an Amnesty International report concerning Qatar for the year 2022. Information on the exact wording can be found in the questionnaire (1). If a respondent had received the neutral sports treatment (see the table below), the probability of agreeing and looking for more information increases significantly. We interpret this as support for the validity of our frames as respondents feel less need to get better informed about the situation in Qatar if receiving either the negative human rights frame or the positive efficiency frame.

	(1)<br>DV: Behavioural outcome
Treatment: neutral	0.10**<br>[0.03,0.16]
Germany	-0.15***<br>[-0.15,-0.15]
Croatia	0.65***<br>[0.65,0.65]
Italy	0.62***<br>[0.62,0.62]
Hungary	-0.25***<br>[-0.25,-0.25]
Romania	0.37***<br>[0.37,0.37]
Poland	0.33***<br>[0.33,0.34]
Sweden	-0.26***<br>[-0.26,-0.26]
Intercept	-0.44***<br>[-0.46,-0.42]
Observations	13441
Pseudo R2	0.02

95% confidence intervals in brackets
* $p < 0.05$, ** $p < 0.01$, *** $p < 0.001$

Table S1. Results of logistic regression with post-stratification weights and with the behavioral outcome as the dependent variable. Standard errors are clustered at the country level.

S2 Confirmatory factor analyses

We extracted the latent variable based on country-specific confirmatory factor analyses using three manifest variables. Running separate models to extract the factors constitutes a more conservative test as it allows for structural differences between countries. In addition, looking at the underlying dimension and not only the three manifest variables separately decreases measurement error. The results are presented below.

	(1) United Kingdom	(2) Germany	(3) Croatia	(4) Italy	(5) Hungary	(6) Romania	(7) Poland	(8) Sweden
Reasonable decision								
Latent variable	1.00	1.00	1.00	1.00	1.00	1.00	1.00	1.00
	[1.00,1.00]	[1.00,1.00]	[1.00,1.00]	[1.00,1.00]	[1.00,1.00]	[1.00,1.00]	[1.00,1.00]	[1.00,1.00]
Intercept	2.98***	1.98***	3.70***	3.51***	3.75***	4.86***	3.53***	1.96***
	[2.88,3.08]	[1.90,2.06]	[3.58,3.82]	[3.40,3.61]	[3.65,3.85]	[4.77,4.96]	[3.43,3.63]	[1.88,2.04]
Good organization								
Latent variable	0.76***	0.87***	0.74***	0.72***	0.99***	0.82***	0.87***	1.21***
	[0.70,0.82]	[0.79,0.95]	[0.66,0.82]	[0.66,0.77]	[0.91,1.07]	[0.74,0.90]	[0.81,0.93]	[1.09,1.33]
Intercept	4.39***	3.85***	5.18***	4.71***	4.68***	5.50***	4.42***	3.35***
	[4.29,4.48]	[3.75,3.95]	[5.09,5.27]	[4.62,4.80]	[4.60,4.77]	[5.41,5.59]	[4.33,4.51]	[3.25,3.45]
Improved reputation								
Latent variable	1.10***	1.36***	0.93***	1.04***	1.01***	0.93***	1.02***	1.31***
	[1.03,1.17]	[1.24,1.47]	[0.85,1.01]	[0.99,1.10]	[0.93,1.09]	[0.86,1.01]	[0.95,1.10]	[1.19,1.44]
Intercept	3.49***	2.50***	4.46***	3.96***	4.67***	5.15***	3.99***	2.65***
	[3.39,3.59]	[2.41,2.58]	[4.35,4.58]	[3.86,4.06]	[4.58,4.76]	[5.06,5.25]	[3.90,4.08]	[2.56,2.75]
var(Latent variable)	2.86***	1.42***	2.46***	3.12***	2.18***	2.24***	2.62***	1.32***
	[2.56,3.15]	[1.18,1.65]	[2.14,2.79]	[2.84,3.39]	[1.92,2.45]	[1.99,2.50]	[2.35,2.90]	[1.09,1.56]
var(Reasonable decision)	1.44***	1.08***	1.39***	1.15***	1.53***	0.90***	1.48***	0.95***
	[1.24,1.65]	[0.93,1.23]	[1.12,1.66]	[0.94,1.37]	[1.29,1.78]	[0.70,1.11]	[1.25,1.72]	[0.81,1.08]
var(Good organization)	1.79***	2.68***	1.21***	1.72***	0.97***	1.06***	1.25***	1.81***
	[1.62,1.96]	[2.50,2.87]	[1.05,1.37]	[1.56,1.88]	[0.80,1.15]	[0.87,1.26]	[1.09,1.42]	[1.58,2.04]
var(Improved reputation)	0.64***	0.36***	1.42***	0.81***	1.02***	1.15***	0.90***	1.04***
	[0.41,0.87]	[0.16,0.56]	[1.16,1.67]	[0.62,1.00]	[0.84,1.20]	[0.95,1.35]	[0.67,1.13]	[0.82,1.26]
Observations	1729	1788	1708	1817	1669	1781	1723	1802

95% confidence intervals in brackets

* $p < 0.05$, ** $p < 0.01$, *** $p < 0.001$

Table S2. Results of country-specific CFAs with post-stratification weights to extract the latent variable used as the main dependent variable.

S3 Main regression results with and without controls

This table presents the main regression results used to calculate the effects shown in Figure 1 (Model 1). The second model adds some control variables as a robustness check.

	(1)	(2)
Intercept	4.32*	3.78*
	[4.27; 4.38]	[3.26; 4.30]
Treatment: negative	−0.49*	−0.46*
	[−0.69; −0.30]	[−0.64; −0.27]
Treatment: positive	0.27*	0.25*
	[0.17; 0.36]	[0.15; 0.35]
Gender: female		0.21*
		[0.06; 0.36]
Gender: other		−0.09
		[−0.76; 0.58]
Age		0.00
		[−0.03; 0.03]
Age (squared)		−0.00
		[−0.00; 0.00]
Education		−0.04
		[−0.12; 0.05]
Political interest		0.02*
		[0.01; 0.03]
Left-right placement		0.08*
		[0.03; 0.13]
Interest in football		0.03
		[−0.00; 0.07]
R^2	0.07	0.09
Adj. R^2	0.07	0.08
Num. obs.	13079	11062
RMSE	1.14	1.13
N Clusters	8	8

95% confidence intervals in brackets

* $p < 0.05$, ** $p < 0.01$, *** $p < 0.001$

Table S3. Model 1: Main regression results post-stratification weights and country dummies. Standard errors clustered at the country level. Figure 1 is based on regression results from Model 1. Model 2: Main model with post-stratification weights, individual-level controls and country dummies. Standard errors clustered at the country level.

S4 Country-specific regression results

For Figure 3 we estimated country-specific regressions and the results are presented below. As we are interested in whether treatment effects vary between countries, this is the most straight-forward way to estimate such effects. However, there are several robustness checks fully underlying the conclusions from this approach.

	(1)<br>United Kingdom	(2)<br>Germany	(3)<br>Croatia	(4)<br>Italy	(5)<br>Hungary	(6)<br>Romania	(7)<br>Poland	(8)<br>Sweden
Treatment: negative	-0.29***	-0.14**	-0.57***	-0.74***	-0.63***	-0.75***	-0.52***	-0.25***
	[-0.45,-0.14]	[-0.24,-0.03]	[-0.73,-0.40]	[-0.89,-0.58]	[-0.76,-0.50]	[-0.90,-0.60]	[-0.66,-0.38]	[-0.35,-0.16]
Treatment: positive	0.34***	0.14*	0.27***	0.47***	0.21**	0.25***	0.24**	0.19***
	[0.18,0.51]	[0.03,0.25]	[0.11,0.43]	[0.31,0.63]	[0.08,0.34]	[0.11,0.38]	[0.09,0.39]	[0.08,0.30]
Intercept	4.22***	4.23***	4.32***	4.32***	4.36***	4.39***	4.33***	4.26***
	[4.11,4.34]	[4.16,4.31]	[4.20,4.44]	[4.21,4.44]	[4.27,4.45]	[4.29,4.49]	[4.23,4.44]	[4.18,4.34]
Observations	1729	1788	1708	1817	1669	1781	1723	1802
R^2	0.04	0.01	0.09	0.13	0.10	0.13	0.06	0.04
Adjusted R^2	0.04	0.01	0.08	0.13	0.10	0.13	0.06	0.04

95% confidence intervals in brackets

* $p < 0.05$, ** $p < 0.01$, *** $p < 0.001$

Table S4. Regression results with post-stratification weights. Figure 3 is based on these regression results.

S5 Additional analyses on media environment effects

We are aware that our cross-country comparison with eight cases does not allow for strong causal claims regarding the role of the information environment. Hence, we added run some additional analyses to further support the plausibility of our interpretation. Figure S1 presents the proportion of respondents unable to assess the human rights situation in Qatar while we distinguish between countries lower and higher media plurality. "Better" media environment increases the probability to be able to assess Qatar.

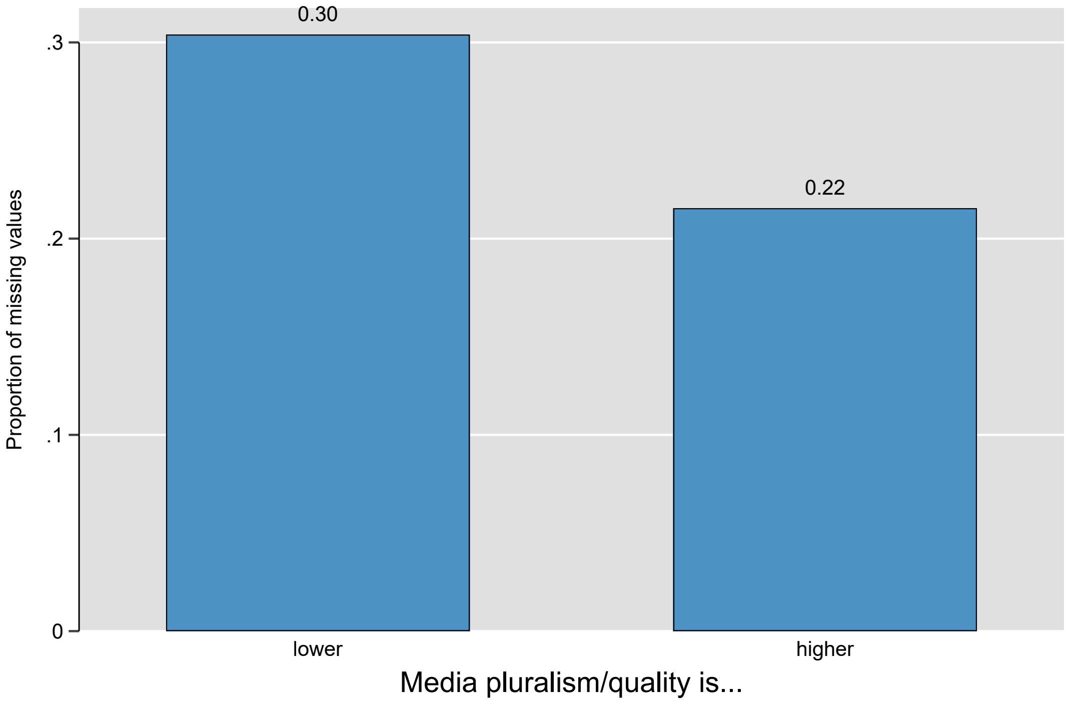

Figure S1. **Proportion of respondents unable to assess the human rights situation in Qatar**. The y-axis plots the share of respondents who indicated that they do not know to or do not want to say how they assess the human rights situation in Qatar. This is done separately for countries with lower (Croatia, Hungary, Poland, and Romania) and higher (Germany, Italy, Sweden, and the United Kingdom) media plurality. Number of valid responses = 15930; post-stratification weights are applied.

On the next page, we present the results of a regression model including an interaction of our treatments as well as the binary measure of being unable to assess (1 = unable to assess). We find that the difference in treatment effects between the positive and the negative treatment are significantly larger for the group of respondents unable to assess the human rights situation in Qatar.

	(1)
Treatment: negative	-0.41***
	[-0.46,-0.35]
Treatment: positive	0.24***
	[0.18,0.30]
Unable to assess	0.42***
	[0.33,0.50]
Treatment: negative x Unable to assess	-0.40***
	[-0.52,-0.28]
Treatment: positive x Unable to assess	0.08
	[-0.03,0.19]
Germany	0.01
	[-0.07,0.09]
Croatia	-0.04
	[-0.13,0.05]
Italy	-0.03
	[-0.12,0.06]
Hungary	-0.08
	[-0.17,0.00]
Romania	-0.06
	[-0.15,0.03]
Poland	-0.02
	[-0.11,0.07]
Sweden	0.00
	[-0.07,0.08]
Intercept	4.25***
	[4.18,4.32]
Observations	14017
R^2	0.09
Adjusted R^2	0.08

95% confidence intervals in brackets

* $p < 0.05$, ** $p < 0.01$, *** $p < 0.001$

Table S5. Regression results for the interaction with a dummy variable for being unable to assess human rights in Qatar. Model with post-stratification weights and country dummies. Standard errors clustered at the country level.

S6 Multi-level model results

As part of the robustness checks, we compare the results from Table S3 with results from a multi-level model. While the low number of higher-level units makes a multi-level model less robust than models including country dummies and correcting the standard errors, we nevertheless want to make sure that model specifications do not influence our findings. As the table shows, this is not at all the case.

Treatment: negative	-0.48***
	[-0.64,-0.32]
Treatment: positive	0.27***
	[0.20,0.34]
Intercept	4.31***
	[4.26,4.35]
var(Country level)	
Intercept	0.00
	[0.00,0.00]
var(Individual level)	
Intercept	1.14**
	[1.04,1.26]
Observations	14017
N Clusters	8

95% confidence intervals in brackets
* $p < 0.05$, ** $p < 0.01$, *** $p < 0.001$

Table S6. Multi-level regression results with post-stratification weights. Note that convergence is achieved but that the remaining country-level variance cannot be estimated properly. This is probably due to it being close to 0.

S7 Regression results with individual manifest measures

In the pre-registration, we suggested to run separate regression models for each of the three manifest variables. While this is of course feasible and informative, we decided to rely on a latent variable representing the three items. However, to show that this has no impact on our conclusions and to follow the pre-analysis plan, the table below presents the respective regression results. There are no substantially relevant differences to our main model.

	Reasonable decision	Good organizer	Improved reputation
Intercept	2.03*	3.50*	2.78*
	[1.93; 2.13]	[3.45; 3.56]	[2.68; 2.88]
Treatment: negative	−0.68*	−0.60*	−0.72*
	[−1.00; −0.35]	[−0.74; −0.46]	[−1.00; −0.43]
Treatment: positive	0.36*	0.33*	0.41*
	[0.24; 0.48]	[0.23; 0.42]	[0.26; 0.55]
Germany	0.00	0.49*	−0.22*
	[−0.01; 0.01]	[0.48; 0.49]	[−0.23; −0.22]
Italy	1.50*	1.34*	1.26*
	[1.50; 1.51]	[1.33; 1.34]	[1.26; 1.27]
United Kingdom	1.02*	1.10*	0.83*
	[1.02; 1.02]	[1.10; 1.10]	[0.83; 0.83]
Croatia	1.69*	1.75*	1.76*
	[1.67; 1.71]	[1.75; 1.76]	[1.74; 1.77]
Romania	2.91*	2.14*	2.46*
	[2.90; 2.92]	[2.14; 2.14]	[2.44; 2.47]
Poland	1.53*	1.03*	1.28*
	[1.52; 1.54]	[1.02; 1.03]	[1.27; 1.28]
Hungary	1.76*	1.29*	1.98*
	[1.75; 1.77]	[1.28; 1.29]	[1.97; 2.00]
Country dummies	Yes	Yes	Yes
R^2	0.23	0.15	0.23
Adj. R^2	0.23	0.15	0.23
Num. obs.	11644	11068	12090
RMSE	1.82	1.75	1.82
N Clusters	8	8	8

95% confidence intervals in brackets

* $p < 0.05$, ** $p < 0.01$, *** $p < 0.001$

Table S7. Regression results with individual manifest measures, post-stratification weights and with country dummies. Reference category: Sweden. Standard errors clustered at the country level.

S8 Logistic regression results

As another test of the potential effect of model specifications, we also run a non-linear regression model for which we recoded the dependent variable (1 = values of the latent variable > 4 .). The table shows that even such a change does not have an effect on our main findings.

	Latent variable (binary)
Treatment: negative	-0.74***
	[-0.93,-0.55]
Treatment: positive	0.36***
	[0.28,0.44]
Germany	-0.33***
	[-0.34,-0.32]
Croatia	0.27***
	[0.26,0.27]
Italy	0.25***
	[0.25,0.25]
Hungary	0.35***
	[0.35,0.35]
Romania	0.43***
	[0.42,0.43]
Poland	0.36***
	[0.35,0.36]
Sweden	-0.05***
	[-0.05,-0.05]
Intercept	0.20***
	[0.13,0.26]
Observations	14017
Pseudo R2	0.05

95% confidence intervals in brackets

* $p < 0.05$, ** $p < 0.01$, *** $p < 0.001$

Table S8. Logistic regression results with post-stratification weights and country dummies. Standard errors clustered at the country level. The dependent variable has been rescaled (1 = values of the latent variable > 4 .)

S9 Alternative test for country heterogeneity

In the table below, we present results for adding interactions of our treatments with the country dummies to identify country heterogeneity. Again, the effects are not substantially different from running separate regression models and the findings concerning country difference seem to be rather robust.

	Latent variable
Treatment: negative	-0.29***
	[-0.45,-0.14]
Treatment: positive	0.34***
	[0.18,0.51]
Germany	0.01
	[-0.13,0.15]
Croatia	0.09
	[-0.07,0.26]
Italy	0.10
	[-0.07,0.26]
Hungary	0.14
	[-0.01,0.29]
Romania	0.17*
	[0.01,0.32]
Poland	0.11
	[-0.05,0.26]
Sweden	0.04
	[-0.10,0.18]
Treatment: negative x Germany	0.15
	[-0.04,0.34]
Treatment: negative x Croatia	-0.27*
	[-0.50,-0.05]
Treatment: negative x Italy	-0.44***
	[-0.66,-0.22]
Treatment: negative x Hungary	-0.34**
	[-0.54,-0.14]
Treatment: negative x Romania	-0.45***
	[-0.67,-0.24]
Treatment: negative x Poland	-0.23*
	[-0.44,-0.01]
Treatment: negative x Sweden	0.04
	[-0.15,0.23]
Treatment: positive x Germany	-0.20*
	[-0.40,-0.01]
Treatment: positive x Croatia	-0.08
	[-0.30,0.15]
Treatment: positive x Italy	0.13
	[-0.10,0.35]
Treatment: positive x Hungary	-0.13
	[-0.34,0.07]
Treatment: positive x Romania	-0.10
	[-0.31,0.11]
Treatment: positive x Poland	-0.11
	[-0.33,0.11]
Treatment: positive x Sweden	-0.15
	[-0.35,0.05]
Intercept	4.22***
	[4.11,4.34]
Observations	14017
R^2	0.08
Adjusted R^2	0.08

95% confidence intervals in brackets
* $p < 0.05$, ** $p < 0.01$, *** $p < 0.001$

Table S9. Regression results with post-stratification weights and country dummies, which are interacted with the treatments.Standard errors clustered at the country level.

S10 Sub-group analyses

While not at the heart of our argument, we test the robustness of our findings by looking at potential individual-level moderators. In other words, while this should not be an issue due to the applied randomization, it could still be of interest to see whether treatment effects differ due to individual-level factors. Specifically, we look at four measures:

Ideological self-placement: Respondents were asked to place themselves on a seven-point left-right scale with lower values indicating a more left-wing position.

Authoritarian values: We use mean values of the KSA-3 Authoritarianism Short Scale (2). High values indicate more authoritarian values.

Interest in football: We use an indicator asking about the respondent's general interest in football on a seven-point scale. Low values indicate lower levels of interest.

Information on the exact wording can be found in the questionnaire (1). The table below presents the regression results in detail.

	(1)	(2)	(3)
Intercept	4.00*	3.26*	4.30*
	[3.78; 4.23]	[2.96; 3.56]	[4.14; 4.45]
Ideological self-placement (left-right)	0.08*		
	[0.03; 0.12]		
Treatment: negative	−0.51*	−0.49*	−0.75*
	[−0.72; −0.30]	[−0.81; −0.17]	[−1.08; −0.42]
Treatment: positive	0.23*	0.34*	0.37*
	[0.09; 0.36]	[0.17; 0.52]	[0.27; 0.47]
Ideology x Treatment: negative	0.01		
	[−0.02; 0.04]		
Ideology x Treatment: positive	0.00		
	[−0.04; 0.05]		
Authoritarianism		0.26*	
		[0.19; 0.32]	
Authoritarianism x Treatment: negative		−0.00	
		[−0.04; 0.04]	
Authoritarianism x Treatment: positive		−0.02	
		[−0.07; 0.03]	
Interest in football			0.01
			[−0.02; 0.04]
Interest in football x Treatment: negative			0.07*
			[0.03; 0.11]
Interest in football x Treatment: positive			−0.03*
			[−0.04; −0.01]
R^2	0.08	0.13	0.08
Adj. R^2	0.08	0.13	0.08
Num. obs.	11136	12972	13039
RMSE	1.13	1.10	1.13
N Clusters	8	8	8

* Null hypothesis value outside the confidence interval.

Table S10. Individual treatment effects with post-stratification weights. Robust standard errors clustered at the country level.

References

[1] S. Hellmeier, H. Giebler, E. Keremoglu, and J. Gerschewski. PAYOFF – sportswashing and the 2022 FIFA world cup in Qatar (survey experiment), 2022. URL https://osf.io/h84an/?view_only=0917472a1480450a9eb07caf778a6880.

[2] D. Nießen, I. Schmidt, C. Beierlein, and C. M. Lechner. Authoritarianism short scale (ksa-3). *Zusammenstellung sozialwissenschaftlicher Items und Skalen (ZIS)*, 2019. doi: 10.6102/zis272. URL https://zis.gesis.org/DoiId/zis272.
